# Visual programming for accessible interactive musculoskeletal models

**DOI:** 10.1186/s13104-022-05994-5

**Published:** 2022-03-22

**Authors:** Julia Manczurowsky, Mansi Badadhe, Christopher J. Hasson

**Affiliations:** 1 Department of Physical Therapy, Movement and Rehabilitation Sciences, Northeastern University, 360 Huntington Avenue, 301 Robinson Hall, Boston, MA 02115-5005 USA; 2 Department of Bioengineering, Northeastern University, Boston, USA; 3 Department of Biology, Northeastern University, Boston, USA

**Keywords:** Musculoskeletal modeling, Myoelectric, Electromyography, Engineering, Neuroscience, Rehabilitation, Visual programming, Simulink

## Abstract

**Objective:**

Musculoskeletal modeling and simulation are powerful research and education tools in engineering, neuroscience, and rehabilitation. Interactive musculoskeletal models (IMMs) can be controlled by muscle activity recorded with electromyography (EMG). IMMs are typically coded using textual programming languages that present barriers to understanding for non-experts. The goal of this project was to use a visual programming language (Simulink) to create and test an IMM that is accessible to non-specialists for research and educational purposes.

**Results:**

The developed IMM allows users to practice a goal-directed task with different control modes (keyboard, mouse, and EMG) and actuator types (muscle model, force generator, and torque generator). Example data were collected using both keyboard and EMG control. One male participant in his early 40’s performed a goal-directed task for four sequential trials using each control mode. For EMG control, the participant used a low-cost EMG system with consumer-grade EMG sensors and an Arduino microprocessor. The participant successfully performed the task with both control modes, but the inability to grade muscle model excitation and co-activate antagonist muscles limited performance with keyboard control. The IMM developed for this project serves as a foundation that can be further tailored to specific research and education needs.

## Introduction

Musculoskeletal modeling and simulation are commonly used as research tools for estimating quantities such as individual muscle forces or bone-on-bone contact forces [1, 2]. A specialty area of modeling employs interactive musculoskeletal models (IMMs), in which a user interacts with the model in a closed-loop fashion (also known as human-in-the-loop models), and the model can be controlled with neural activity recorded from the surface of muscles via electromyography (EMG). In addition to testing sensorimotor control hypotheses [3–6], IMMs have been used in the control of prosthetics [7, 8] and exoskeletons [9, 10]. IMMs may also be helpful in education, such as enhancing a student’s understanding of how the nervous system coordinates a redundant musculoskeletal system.

Many IMMs are written using textual programming languages such as C++ [11–13], which non-experts may find opaque. Visual programming languages (VPL) are an alternative that uses blocks to represent code functions and arrows to represent function inputs and outputs. This allows the visual structure of the program to mimic a conceptual flowchart, thereby facilitating understanding of program function by non-experts. However, there are few existing examples of VPL-based musculoskeletal models, and most implementations are not IMMs.

An early VPL-based musculoskeletal model used Simulink (MathWorks, Natick, MA) to create virtual muscles that could be used to simulate human movements [14]. Others have used Simulink-based musculoskeletal models to simulate gait [15–18] and arm movements [17, 18]. Although Simulink has been used for online control applications with hard or soft exosuits, it is mainly used to control the low-level robotic hardware [19, 20], with the musculoskeletal model implemented in a textual language such as C++ [19]. Thus, there is a need to develop a VPL-based IMM that allows human-in-the-loop control, which can be used by non-specialists for research and educational purposes.

The goal of this project was to create a VPL-based user-friendly IMM, which can serve as research and education tools in a variety of domains. For example, a neuroscientist may use the IMM to understand how the nervous system adapts to changes in the mechanical properties of a prosthetic arm, or a physical therapist may use the IMM to study how different neuromuscular impairments affect movement control.

## Main text

### Methods

#### Overview

The VPL Simulink (R2021a) was used to program an IMM of a human arm for use in education and research. The specific IMM design criteria included: (1) human-in-the-loop control with either a keyboard, mouse, or EMG, (2) different modes of actuation (muscle model, force generator, or torque generator), (3) capability to perform a goal-directed task, and (4) ability to save relevant variables for further analysis. Only those model components essential for simulating human motion were included. The rationale was to minimize the number of model parameters and make it simpler for users to understand the effects of parameter changes on the behavior of the arm.

#### Details

**Fig. 1:**
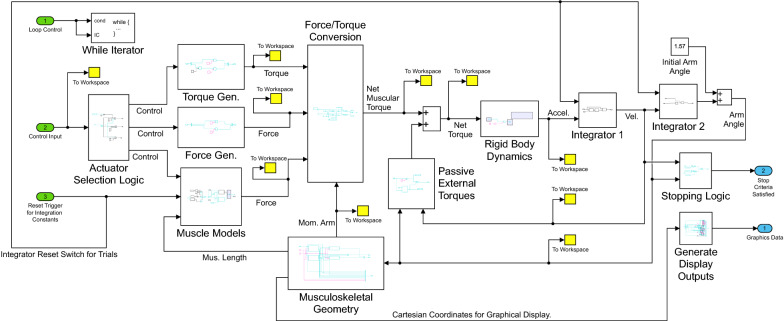
Simulink model diagram showing main program components for the interactive musculoskeletal model

**Fig. 2:**
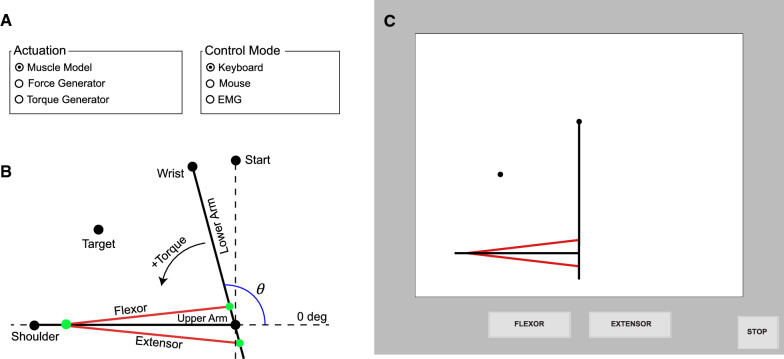
**A** Available options for model actuation and user control type (EMG = electromyographic control). **B** Schematic showing geometry and key components of the virtual arm. **C** Graphical user interface

The musculoskeletal model is of the same form as previously used by our group [21, 22]. The Simulink model contains several discrete components, including actuator models, rigid body dynamics and musculoskeletal geometry, and integrators (Fig. 1). These components were embedded in a loop that allows multiple trials to be performed for motor adaptation and learning experiments (this higher-level loop is not shown in Fig. 1). Simulink dashboard elements make a simple user-interface with radio buttons (Fig. 2A) for selection of control (keyboard/mouse/EMG) and actuation (torque/force/muscle) modes. The musculoskeletal model is planar with a fixed upper arm and mobile lower arm, both modeled as rigid links, connected by a hinge joint (Fig. 2B). A custom visual display shows the virtual arm, task features, and control buttons for mouse control (Fig. 2C). A Level-2 MATLAB S-Function generates the display and collects user control inputs. This is the only custom S-Function; all other operations are performed with native Simulink blocks. Although more sophisticated models could be created with products like Simscape Multibody (Mathworks), this was avoided to maximize accessibility, so users only need a single software package (MATLAB/Simulink). A Runge–Kutta solver was used with a fixed-step size of 1/150 s. This represented a tradeoff between simulation speed and integration accuracy on a Windows 10 laptop with an Intel Core i7-7700HQ CPU @ 2.8 GHz with 32 GB of RAM (for higher accuracy or patient-critical applications, a real-time operating system and smaller step size would be preferred for the mathematically stiff IMM).

#### Actuation modes

The model allows three different modes of actuation. In the torque generator mode, the control signals are mapped directly into elbow flexor and extensor torques. In the force generator mode, control signals are mapped into actuator forces and multiplied by muscle moment arms to create joint torques. In the muscle model mode, muscle mechanics are included using classic Hill-type two-element muscle models, with each comprised of a contractile element in series with an elastic element. The force produced by the contractile element depends on its activation, length, and velocity. The elastic component has a nonlinear stiffness; it’s compliant at low forces and stiffens at higher forces (muscle model details are in Hasson [21]). As for the force generators, the muscle model forces are multiplied by moment arms to produce flexor and extensor muscular torques on the elbow joint. The user can change any of the actuator properties through a Simulink dashboard interface.

#### Control modes

Users can control the model with their own muscles using an EMG system (a low-cost system is used here; see “Experimental Protocol” section for details). EMG measured from the user’s elbow flexors and extensors serves as excitation signals to the corresponding flexor and extensor muscle models. EMG control is proportional, i.e., users can vary the magnitude, timing, and duration of the actuator control signals. Alternative control modes allow users to either click on buttons or use keystrokes with the keyboard (left/right arrows). These modes only allow “bang-bang” control, i.e., the excitation level is fixed, and the user can only vary the timing and duration of the control signals.

#### Actuator geometry

The force generators and muscle models share the same geometry. They originate on the proximal end of the upper arm segment and insert on the proximal end of the lower arm segment (Fig. 2B). This loosely mimics the attachments of the elbow flexors (short head of the biceps brachii, brachialis, and brachioradialis) and elbow extensors (triceps brachii). For simplicity, the moment arms of the actuators were calculated based on the straight-line geometry. If desired, more realistic values could be set based on more complex anatomical models, such as those available in OpenSim [23].

#### Passive components

Two passive torques were added to the active torque produced by the actuators. An exponential ligamentous torque prevents the arm from moving beyond a physiological range of motion, emulating the action of elbow ligaments (and bony endpoints). A damping torque makes the experimental task more manageable, which requires stopping the arm on a spatial target (otherwise, the frictionless environment makes stopping difficult).

#### Motor task

The Simulink model allows users to practice a goal-directed task with the IMM. The task is to move the virtual arm from a starting position at 90° (Fig. 2C) to a target position 45° away (counterclockwise/elbow flexion) as fast as possible, with a movement time limit of 5 s. Users can perform any number of trials. A trial is considered successful when kinematic criteria are satisfied (arm angle within ± 2° of target and arm angular velocity below 0.001°/s). When the trial ends (successfully or timed out), the arm automatically resets back to the starting position, and the user begins another trial.

#### Experimental protocol

To illustrate the performance of the IMM, example data were collected using both keyboard and EMG control with the muscle models. One male participant in his early 40’s performed the task for four sequential trials using each control mode. To maximize accessibility, a low-cost EMG system was used with consumer-grade EMG sensors (MyoWare, Advancer Technologies, LLC). The sensors integrated bipolar snap-electrode receptacles spaced 3 cm apart with a third separate reference snap-electrode receptacle with a 6 cm wire lead (round pre-gelled disposable snap electrodes were used; Kendall ECG Electrodes, Covidien, Dublin, Ireland). The participant’s skin was prepared by rubbing with an abrasive gel and cleaning with alcohol. One EMG sensor was placed on the center of the biceps muscle belly and the other on the center of the triceps muscle belly (lateral head); both sensors were oriented parallel to the muscle fibers. After placement, the electrodes were covered with elastic pre-wrap (Muller Sports Medicine, Prairie Du Sac, Wisconsin). The EMG sensors included onboard electronics that amplified the measured differential voltage, applied a high-pass filter (to remove the offset), full-wave rectified the signal, and then produced a linear envelope with an integration amplifier. The analog linear envelopes from the biceps and triceps were digitized by a 10-bit Arduino microprocessor, which had an onboard FTDI FT232RL USB-to-TTL serial communication chip (Nano v3.3, Arduino SA, Lugano, Switzerland). The digitized EMG signals were acquired through a USB connection (the FTDI drivers provided a virtual COM port) using the MATLAB serialport.m function in the Level-2 S-Function that provided the graphical user interface. To enhance safety, a USB isolator (Adafruit Industries) was placed between the Arduino and PC USB port.

## Discussion

**Fig. 3:**
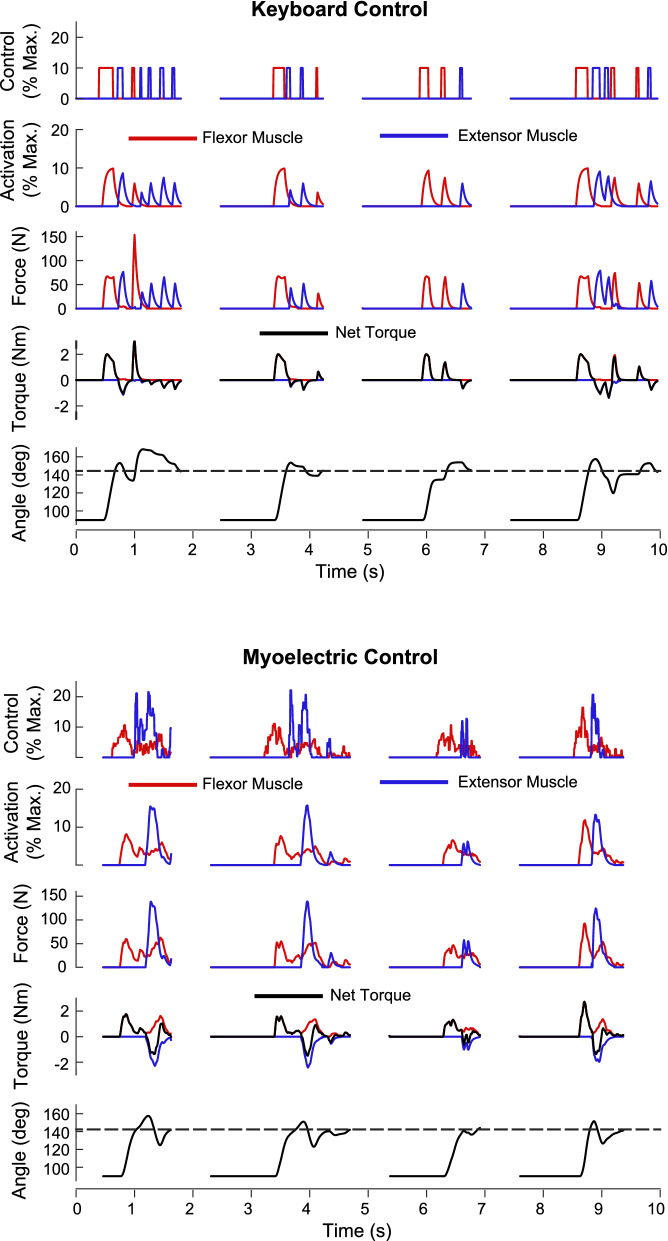
Example data from one participant that performed a goal-directed task with an interactive musculoskeletal model of an arm using keyboard and myoelectric control. Four sequential trials are shown. The goal was to move the arm model from a starting position to a target (horizontal dashed line in the arm angle plots) as quickly as possible

The VPL-based IMM allows users to control a virtual arm using different control and actuation modes. An example contrasting keyboard and EMG control using muscle model actuation is shown in Fig. 3. The disadvantages of keyboard control are evident as the participant only had control over the timing and duration of the muscle excitation. On the other hand, myoelectric control allows excitation amplitude to be graded and affords the ability to co-activate flexor and extensor muscle models, which may help in stabilizing the arm near the target. While this example shows some of the variables that can be viewed and analyzed by program users, other variables can also be plotted, including those related to the internal workings of the muscle models, such as the contractile and elastic element lengths and velocities and muscle moment arms. Using Simulink, these variables populate the MATLAB workspace, and a simple MATLAB script can perform post-processing of the data (as was done in this case). Simulink scope blocks can also be inserted directly into the Simulink model to view signals during program execution.

The IMM can serve as both an education and research tool for a wide audience. For example, it could serve as an introduction to musculoskeletal modeling and myoelectric control for an engineering student. The block diagram would facilitate understanding of model function. Components could be altered to simulate a myoelectric prosthesis. Alternatively, a neuroscientist might be interested in understanding how the nervous system adapts to changes in musculoskeletal dynamics, such as a change in musculoskeletal geometry. On the other hand, a physical therapist could operate the model for exposure to the internal and dynamical complexities that impact movement production with some basic instruction. Various disorders could be simulated. For example, the therapist could experience how a reduction in strength impacts goal-directed actions. The interactive, user-modifiable, myoelectrically-driven, upper-limb musculoskeletal model developed by this project serves as a foundation that can be further tailored to specific research and education needs. We made the IMM available with a permissive open-source software license to maximize accessibility (see link in “Availability of data and materials” sections).

## Limitations

The program was designed to run in the basic Simulink programming environment (without add-ons such as Simscape Multibody) on a standard Windows PC or laptop. This increases ease of access to the source code so that users can change the block diagram and interactively experience the effects. However, running the program in this way on a non-real-time operating system limits execution speed, which could be increased by using a real-time operating system or deploying the program on a dedicated microprocessor. For accessibility, a low-cost EMG system was used, and the musculoskeletal model has a relatively simple architecture: a planar model with lumped flexor and extensor muscle models with straight-line geometry. For some research applications, a more detailed model architecture and higher-grade EMG system may be desired.

## Data Availability

The datasets used and analyzed during the current study are available from the corresponding author on reasonable request. The source code for the interactive musculoskeletal model is freely available on GitHub (https://github.com/cjhasson/simulink-virtual-arm).

## Abbreviations

IMM: Interactive musculoskeletal model
VPL: Visual programming language
EMG: Electromyography
